# Whole‐exome sequencing of a Saudi epilepsy cohort reveals association signals in known and potentially novel loci

**DOI:** 10.1186/s40246-022-00444-6

**Published:** 2022-12-20

**Authors:** Abdulrahman H. Al Anazi, Ahmed S. Ammar, Mahmoud Al-Hajj, Cyril Cyrus, Danah Aljaafari, Iname Khoda, Ahmed K. Abdelfatah, Abdullah A. Alsulaiman, Firas Alanazi, Rawan Alanazi, Divya Gandla, Hetal Lad, Samar Barayan, Brendan J. Keating, Amein K. Al-Ali

**Affiliations:** 1 Department of Neurosurgery, King Fahd Hospital of the University, Alkhobar, College of Medicine, Imam Abdulrahman Bin Faisal University, Dammam, Saudi Arabia; 2 Department of Neurosurgery, King Fahd Hospital, Alhafof, Saudi Arabia; 3 Department of Clinical Biochemistry, College of Medicine, Imam Abdulrahman Bin Faisal University, P. O. Box 1982, 31441 Dammam, Saudi Arabia; 4 Department of Neurology, King Fahd Hospital of the University, Alkhobar, College of Medicine, Imam Abdulrahman Bin Faisal University, Dammam, Saudi Arabia; 5 Department of Surgery, University of Pennsylvania School of Medicine, Philadelphia, PA USA

**Keywords:** Epilepsy, Neurological conditions, WES, Saudi Arabia, Variants

## Abstract

**Background:**

Epilepsy, a serious chronic neurological condition effecting up to 100 million people globally, has clear genetic underpinnings including common and rare variants. In Saudi Arabia, the prevalence of epilepsy is high and caused mainly by perinatal and genetic factors. No whole-exome sequencing (WES) studies have been performed to date in Saudi Arabian epilepsy cohorts. This offers a unique opportunity for the discovery of rare genetic variants impacting this disease as there is a high rate of consanguinity among large tribal pedigrees.

**Results:**

We performed WES on 144 individuals diagnosed with epilepsy, to interrogate known epilepsy-related genes for known and functional novel variants. We also used an American College of Medical Genetics (ACMG) guideline-based variant prioritization approach in an attempt to discover putative causative variants. We identified 32 potentially causative pathogenic variants across 30 different genes in 44/144 (30%) of these Saudi epilepsy individuals. We also identified 232 variants of unknown significance (VUS) across 101 different genes in 133/144 (92%) subjects. Strong enrichment of variants of likely pathogenicity was observed in previously described epilepsy-associated loci, and a number of putative pathogenic variants in novel loci are also observed.

**Conclusion:**

Several putative pathogenic variants in known epilepsy-related loci were identified for the first time in our population, in addition to several potential new loci which may be prioritized for further investigation.

**Supplementary Information:**

The online version contains supplementary material available at 10.1186/s40246-022-00444-6.

## Background

Epilepsy is a group of chronic neurological diseases characterized by unprovoked seizures due to abnormal neuronal firing in the brain. It is one of the most frequently encountered medical problems in neurology clinics. Epilepsy affects up to 1 in 26 individuals in the USA and approximately 5–7 per 1000 individuals worldwide with reports indicating that at least 50 to 100 million people in the world suffer from this disease, with approximately 85% of these cases living in developing countries [1–3]. However, 20–30% of all epilepsy cases are due to acquired factors, such as infection, stroke, trauma, neoplasms, and autoimmunity, while the remaining cases are thought to be due to genetic factors [4]. Epidemiological studies have observed an increased risk of epilepsy development in the relatives of epileptic individuals [5].

Genome-wide association studies (GWAS) of epilepsy phenotypes using genome-wide genotyping (GWG) arrays have discovered many single-nucleotide polymorphisms (SNPs) associated with the disease. A meta-analysis conducted by the International League Against Epilepsy in 2014 identified SNPs associated with the development of epilepsy in the *sodium voltage-gated channel alpha subunit 1* (*SCN1A)*, *Protocadherin 7* (*PCDH7*), *FA Complementation Group L (FANCL)* and *Vaccinia Related Kinase 2 (VRK2*) genes [6]. A subsequent large GWAS encompassing over 15,200 epilepsy subjects and 29,600 controls revealed 16 genome-wide significant signals reaching statistical significance. Genes in these loci have diverse roles in histone modification, ion-channels, pyridoxine metabolism, synaptic transmission as well as transcription factors. Interestingly, functional annotation of almost 500 SNPs, that were observed to be significant at a genome-wide statistical threshold, found that most are in intronic (46%) or intergenic (29%) regions, with only 4 non-synonymous SNP associations observed, of which 2 were missense variants [7]. Furthermore, approximately half of the SNPs were implicated to impact gene transcription from gene regulation database analyses [7].

Second-generation sequencing technologies including whole-exome sequencing (WES) have been used in the diagnostic or research settings to identify genetic mutation(s) which may cause highly variable phenotypic expression in epileptic subjects [8–10]. The very nature of the WES approach favors detection of rarer highly penetrant gene-coding variants which are often hard to find in GWG-based GWAS approaches.

Performing WES in Saudi Arabian epilepsy populations offer a unique opportunity for the discovery of rare genetic variants impacting this disease as there is a high rate of consanguinity among large tribal pedigrees. A study in the Eastern province of Saudi Arabia has reported the prevalence of epilepsy to be around 6–7 per 1000 individuals [11]. In the present study, WES was performed on 144 individuals diagnosed with epilepsy with varying age-of-onset. An American College of Medical Genetics (ACMG) guideline-based variant prioritization approach followed WES which allowed for the discovery of potentially causative variants in our cohort of epilepsy subjects.

## Materials and methods

### Patient sampling and ethical approval

Over a period spanning 2018–2020, samples and data from consecutive subjects with epilepsy attending the Neurosurgery Clinics, King Fahd Hospital of the University, Al-Khobar, and King Fahd Hospital, Alhafof, Saudi Arabia, were collected for inclusion in this study. Participants ranged in age from 13–51 and were clinically diagnosed with epilepsy at the point of recruitment. The phenotype data of all subjects were reviewed by a consultant committee to verify uniformity among sites and eligibility consistent with International League Against Epilepsy [12]. In this study, the diagnosis of epilepsy was made by a consultant specializing in epilepsy based on the patients’ clinical history. Moreover, cases with moderate-to-severe intellectual disability, or cancer, were excluded from the study. If there was doubt regarding a patient’s phenotypic eligibility, the individual cases were reviewed by the consultant committee and if needed, additional data were requested prior to a decision being made by the committee regarding inclusion of the patient. However, on completion of the project the medical records of all patients were reviewed again. Among all our epileptic patients included in the study, eight patients also had type 2 diabetes, five patients had hypertension, one patient had cardiovascular disease and stroke, one patient had polycystic ovarian syndrome, and two patients had muscular dystrophy. Table 1 outlines the subjects’ demographic and clinical characteristics.

**Table 1:** Clinical and demographics characteristics of the 144 Saudi epilepsy subjects

Parameter	Value
Age (mean ± SD) (years)	28.30 ± 11.15
Gender (male %)	50.5
Age of initial diagnosis (years)	13–51
Duration of the disease (years)	13.46 ± 8.70
Percentage of subjects with affected family members (%)	24.5
Management of disease	
Monotherapy (%)	57.5%
Poly-therapy (%)	42.5%

Ethical approval for the study was obtained from the local Institutional Review Board (IRB) committees (IRB**-**2015-01-063), and the study was conducted according to the ethical principles of the Declaration of Helsinki and Good Clinical Practice guidelines. All patients included in the study signed a written informed consent.

### DNA sequencing, read alignment, variant calling and quality control

Blood samples were collected from subjects in EDTA vacutainers and after collection were immediately stored at − 80 °C. Standard DNA preparation was performed using DNeasy Blood kits (Qiagen, MD, USA). Whole-exome sequencing libraries were generated using the Agilent SureSelect Human All Exon Kit V5 (Agilent, CA, USA) and sequenced on a HiSeq 2500 instrument (Illumina, CA, USA) using standard paired-end sequencing protocol. Raw sequencing reads were stored as FASTQ files and then aligned to the human reference genome (GRCh37) using Illumina’s Dynamic Read Analysis for GENomics (DRAGEN) Pipeline. Resultant BAM files were position-sorted and duplicate reads marked. Single-sample gVCF files were generated by the DRAGEN Germline Pipeline, and joint calling of all samples in the study cohort were performed by DRAGEN Joint Genotyping.

### Principal components analysis (PCA) and Kinship

KING was used for relatedness inference based on the genotype of exome SNPs (MAF > 0.01) [13]. Estimated kinship coefficient and number of SNPs with zero shared alleles (IBS0) between a pair of individuals were plotted. Parent–offspring, sibling pairs, and unrelated pairs can be distinguished as separate clusters on the scatterplot. Ancestry and kinship toolkit (AKT) was used to calculate PCAs and plot the results [14].

### Variant annotation, filtering and prioritization

Variants were annotated with SnpEff to predict the effects of variants [15]. Rare variants were defined as minor allele frequency (MAF) < 1% in the Genome Aggregation Database (GnomAD) [16]. Intronic, synonymous, 3’ and 5’ UTR, up- and downstream variants were identified and excluded from the analysis. The remaining rare variants were considered to be potentially deleterious variants. Genetic variants classified in ClinVar as “Likely pathogenic” or “Pathogenic”, and in Human Gene Mutation Database (HGMD) as disease-causing mutations (DM) for epilepsy or seizures were collected and curated together with research literatures to server as the knowledgebase for variant prioritization and classification [17, 18].

## Results

### Principal component analysis

**Fig. 1:**
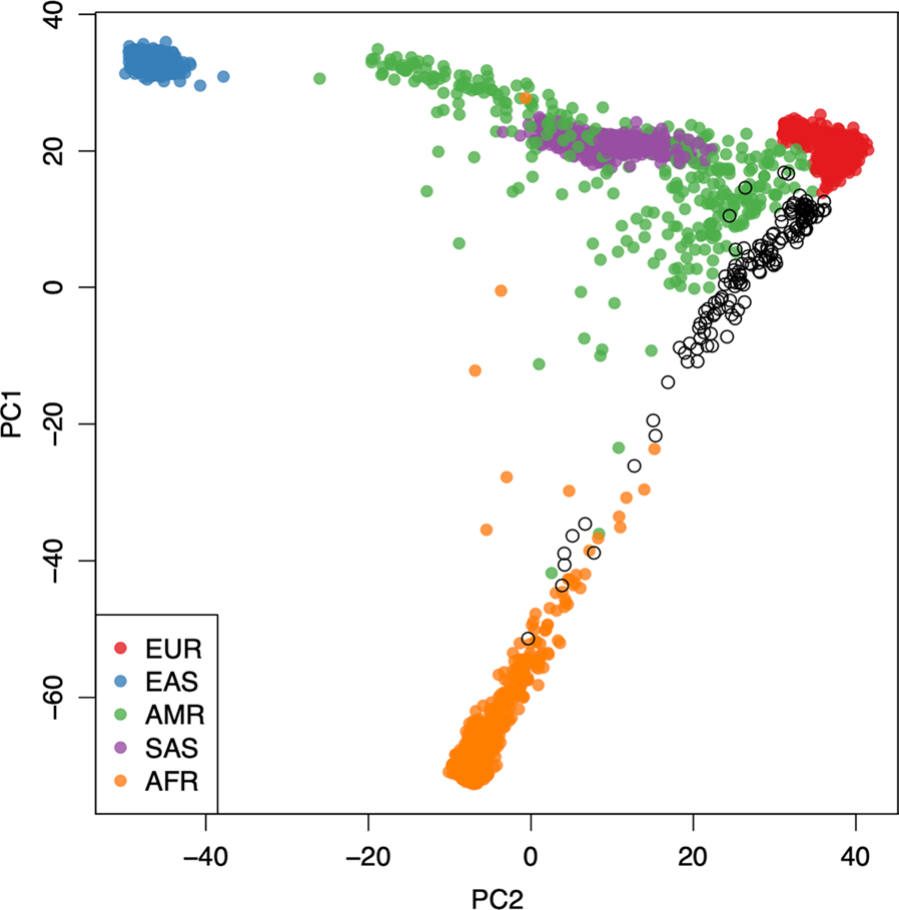
The x-axis and y-axis denote the value of two components of PCA (PC1, PC2), with each dot in the figure representing one individual. The color for individuals belonging to the Epilepsy Disease study group is illustrated in black. The color for individuals from 1000 genome projects, Europeans (EUR), East Asians (EAS), Admixed Americans (AMR), South Asians (SAS), and Africans (AFR) is red, blue, green, purple and orange, respectively

The common genetic variants of these Saudi epilepsy individuals show a unique cluster when compared to the world’s major populations based on principal component analysis (Fig. 1). The samples demonstrated a genetically matched background which avoided false attribution of associations due to population stratification.

### Potentially pathogenic rare variants identified in 44 epilepsy subjects

Based on a combination of variant filtering, and integration of variant databases in ClinVar, and HGMD, we identified a total of 32 potentially causative pathogenic variants across 30 different genes in 44/144 (30%) epilepsy subjects as shown in Table 2. Additional file 1: Supplementary Table S1 outlines additional minor allele frequencies in additional databases and further annotation of these putative pathogenic variants.

**Table 2:** Potentially causative pathogenic variants derived from screening of 144 Saudi epilepsy subjects. Chromosomal position is outlined for the 32 putative pathogenic variants in 30 gene regions along with annotation of the putative pathogenic variant mapped to human reference genome build 37 (GRCh37). Minor allele frequencies (MAF) are shown for the: Saudi epilepsy cohort; Genome Aggregation Database (GnomAD); and Human Gene Mutation Database (HGMD). ClinVar annotation for likely clinical significance is also listed

Chromosome position	Patient(s) with Heterozygous or Homozygous** Variant	Variant Type	Gene (NCBI Gene ID)	Cohort Variant MAF*	ExAC MAF	GnomAD MAF	HGMD Mutation Phenotype	Clinical significance in CLINVAR database
chr1: 43907014	065	Missense	*SZT2*	0.003	0.00034	0.002581	Epileptic encephalopathy early-onset	Conflicting interpretations of pathogenicity
chr1: 47746675	094, 128, 134	Missense	*STIL*	0.01	0.00213	0.003538	Intellectual disability seizures microcephaly	Conflicting interpretations of pathogenicity
chr1: 97915614	101, 141	Splicing	*DPYD*	0.007		0.007067	Dihydropyrimidine dehydrogenase deficiency	drug response
chr1: 119683231	070, 129, 135	Missense	*WARS2*	0.01		0.006159	Intellectual disability	Conflicting interpretations of pathogenicity
chr1: 160011671	021, 045	Missense	*KCNJ10*	0.007		0.001179	Developmental delay failure to thrive ataxia hypotonia and tonic–clonic seizures	Uncertain significance
chr2: 166243269	019	Missense	*SCN2A*	0.003	0.00073	0.005002	Epileptic encephalopathy	Conflicting interpretations of pathogenicity
chr2: 166848930	069	Missense	*SCN1A*	0.003	0.00056	0.004116	Intractable epilepsy	Conflicting interpretations of pathogenicity
chr2: 167138296	085	Missense	*SCN9A*	0.003	0.00189	0.008274	Febrile seizures	Conflicting interpretations of pathogenicity
chr3: 38739016	007, 039	Frame shift	*SCN10A*	0.007	0.0001	0.000130	Refractory epilepsy & autism spectrum disorder	Uncertain significance
chr3: 132378559	009, 101	Stop gained	*UBA5*	0.007		0.009118		Pathogenic
chr4: 119736287	032, 141	Missense	*SEC24D*	0.007		Not seen	Intellectual disability and epilepsy	Likely pathogenic
chr8: 1719594	071	Missense	*CLN8*	0.003	0.00082	0.002221	Neuronal ceroid lipofuscinosis late infantile	Conflicting interpretations of pathogenicity
chr10: 79396648	017**	Stop gain	*KCNMA1*	0.008		Not seen	Epilepsy	
chr11: 9225637	047	Frame shift	*DENND5A*	0.003		Not seen	Epileptic encephalopathy	Pathogenic
chr11: 93521218	087	Missense	*MED17*	0.003		0.000115	Seizures and hypoplasia of the corpus callosum	Uncertain significance
chr12: 42863325	083	Missense	*PRICKLE1*	0.003	1.00E−05	0.000641	Progressive myoclonus epilepsy-ataxia syndrome	Likely pathogenic
chr12: 52164462	096	Missense	*SCN8A*	0.003	1.00E−05	0.000109	Paroxysmal kinesigenic dyskinesia	Conflicting interpretations of pathogenicity
chr12: 111856571	021, 037, 043, 138	Missense	*SH2B3*	0.014	0.00099	0.004796	Erythrocytosis idiopathic	Uncertain significance
chr13: 100925464	048	Missense	*PCCA*	0.003		0.000976	Epilepsy & neurodevelopmental delay	Conflicting interpretations of pathogenicity
chr15: 23006299	002, 009, 040, 044, 050, 057, 089	In-frame insertion	*NIPA2*	0.024		0.008426	Childhood absence epilepsy	
chr15: 52632432	121	Missense	*MYO5A*	0.003		Not seen	Developmental delay seizures & dystonia	Pathogenic
chr15: 73617728	127	Missense	*HCN4*	0.003		0.000018	Myoclonic epilepsy benign infantile	
chr15: 89866693	049	Missense	*POLG*	0.003	0.00036	0.001169	Depression ataxia and cardiomyopathy	Conflicting interpretations of pathogenicity
chr15: 89870429	030	Missense	*POLG*	0.003	0.00044	0.002358	Progressive external ophthalmoplegia	Conflicting interpretations of pathogenicity
chr15: 89876827	032	In-frame insertion	*POLG*	0.003		0.004516	Hepatic encephalopathy	Benign/Likely benign
chr16: 150392	112	Missense	*NPRL3*	0.003		0.000933	Focal epilepsy	Uncertain significance
chr16: 28500627	104	Missense	*CLN3*	0.003	4.00E−05	0.000203	Retinal degeneration	Uncertain significance
chr17: 61791402	084	Missense	*STRADA*	0.003		Not seen		Pathogenic
chr19: 14024452	071	Splicing	*CC2D1A*	0.003		Not seen	Autism spectrum disorder intellectual disability and seizures	Pathogenic
chr20: 61981924	055, 098, 106, 121	Missense	*CHRNA4*	0.014		Not seen	Epilepsy nocturnal frontal lobe	Pathogenic
chr21: 34003387	141	Missense	*SYNJ1*	0.003	0.00082	0.008603	Parkinson disease	Likely benign
chr22: 32188751	019	Stop gained	*DEPDC5*	0.003		Not seen	Epilepsy familial focal with variable foci	Pathogenic

Chromosome position is based on build 37 of the human genome. ExAC = Exome Aggregation Consortium; MAF = Minor allele frequency; GnomAD = Genome Aggregation Database; HGMD = Human Gene Mutation Database

** homozygous variants

The 30 genes harboring the likely pathogenic variants observed in these 44 Saudi epilepsy subjects were then assessed for overlap with 102 previously collated monogenic epilepsy genes [7]. Likely pathogenic variants in 12 of these 102 monogenic epilepsy genes were observed in 44 epileptic subjects: *CHRNA4, CLN3, CLN8, DEPDC5, KCNJ10, KCNMA1, POLG, PRICKLE1, SCN1A, SCN2A, SCN8A* and *SCN9A*. Of the 18 additional genes from Table 2 with likely pathogenic variants, a number including *SZT2*, *SCN10A*, *UBA5* have been reported in whole-exome sequencing in epilepsy subjects [19, 20]. Only one homozygous mutation, a stop-gain, was observed in single individual [21], in *Potassium Calcium-Activated Channel Subfamily M Alpha 1* (*KCNMA)*, a calcium-sensitive potassium channel gene which has been shown to have a role in general and early-onset epilepsy-related phenotypes [22–26]. This individual, a female who was diagnosed with childhood epilepsy at 12 years old, has one family member with a diagnosis of epilepsy and was assessed to have first degree of consanguinity.

### Most commonly observed likely pathogenic variants

An in-frame insertion mutation in *non-imprinted in Prader–Willi/Angelman syndrome region protein 2* (*NIPA2)* (chr15, GRCh37 position: 23006299) was observed in seven of the study subjects (shown in Table 3 along with age of onset). A missense variant in *SH2B Adaptor Protein 3 (SH2B3)* (chr12, position: 111856571) and in *Cholinergic Receptor Nicotinic Alpha 4 Subunit (CHRNA4)* (chr20 position: 61981924) were both observed in four individuals. Missense variants in *STIL Centriolar Assembly Protein (STIL)* (chr1, position: 47746675), and in *Tryptophanyl-TRNA Synthetase 1 (WARS2)* were both observed in three individuals.

**Table 3:** Most commonly observed putative pathogenic variants from whole-exome sequencing across 144 Saudi epilepsy patients

Mutation type, gene and position	Study subject	Age of onset	Gender	Other family members affected	Management	Response to treatment
In-frame insertion in *NIPA2* Pathogenic p.N334_E335insD (chr15: 23006299)	002	7	M	No	Mono	Yes
	009	16	M	No	Mono	Yes
	040	2	F	No	Poly	Partial
	044	24	M	Yes	Mono	Yes
	050	15	F	No	Mono	Yes
	057	1	M	No	Poly	Partial
	089	15	M	Yes	n/a	n/a
Missense variant in *SH2B3* Pathogenic c.622G > C p.Glu208Gln (chr12: 111856571)	021	n/a	M	No	Mono	Yes
	037	8	F	No	Poly	Yes
	043	13	F	No	Mono	Yes
	138	26	F	No	Poly	Yes
Missense variant in *CHRNA4* Pathogenic c.839C > T p.Ser280Phe (chr20: 61981924)	055	31	F	Yes	n/a	n/a
	098	13	M	n/a	n/a	n/a
	106	n/a	F	n/a	n/a	n/a
	121	5	F	No	Mono	Yes
Missense variant in *STIL* Pathogenic c.1455G > C p.Leu485Phe (chr1: 47746675)	094	23	M	No	n/a	n/a
	128	19	M	No	Poly	Yes
	134	15	F	Yes	Mono	Yes
Missense variant in *WARS2* Pathogenic c.37 T > G p.Trp13Gly (chr1: 119683231)	070	22	M	n/a	n/a	n/a
	129	16	M	No	Poly	Yes
	135	9	M	Yes	Poly	Yes

### Variants of unknown significance identified in epilepsy 133 subjects

In highly curated genomic disease databases such as ClinVar, there are a large number of variants of unknown significance (VUS), where there is unknown or conflicting clinical significance to date for association of such variants with epilepsy-related phenotypes. We identified 232 variants of unknown significance across 101 different genes in 133/144 (92%) subjects as shown in Additional file 2:Supplementary  Table S2. Interestingly when the genes harboring these variants of unknown significance were intersected with the 102 previously collated monogenic epilepsy genes it was observed that 43 of these monogenic gene variants were enriched in the 101 loci with VUS from these 133 Saudi epilepsy subjects (Additional file 2: Table S2). We note that two individuals were observed to have homozygous potential pathogenic variants. One individual showed homozygosity for a missense variant in *Spermatogenesis Associated 5 (SPATA5),* with 3 individuals showing heterozygosity for this mutation (Additional file 2: Supplementary Table S2). *SPATA5* has shown clear association with severe childhood epilepsy [27–32]. This female individual was diagnoses with childhood epilepsy at the age of 10 years old and two family members having a diagnosis of epilepsy, and both parents are listed as cousins.

Another homozygous mutation was observed in one individual for a missense mutation in *Calcium Voltage-Gated Channel Subunit Alpha1 H (CACNA1H)* with three additional individuals also carrying one copy of this mutation. Mutations in this gene have been associated with generalized and severe epilepsies [33, 34], although it has been debated whether this is a *bona fide* monogenic epilepsy gene [35]. This female individual was diagnosed with epilepsy at 15 years of age and does not have any other family members with a diagnosis of epilepsy, or any consanguinity noted.

## Discussion

We performed the first whole-exome sequencing study in Saudi Arabia epilepsy subjects. Using 144 individuals, we compared putative pathogenic variants as well as variants of unknown significance with population-based whole-exome sequencing and whole genome sequencing databases.

The highest number of observed mutations across the 144 subjects were observed in *NIPA2*, a highly selective magnesium transporter. This in-frame insertion variant (NP_001171818_.1 p. (Asn334_Glu335insAsp_)) was observed in 7 subjects from 144 overall (5%). This variant has been previously reported within a population of subjects with childhood absence epilepsy (CAE) [36–38].

A missense variant in *CHRNA4* was observed in 4 subjects. *CHRNA4* is a nicotinic acetylcholine receptor, belonging to a superfamily of ligand-gated ion channels which play an established role in signal transmission at synapses. Mutations in *CHRNA4* have been reported with nocturnal frontal lobe epilepsy type 1. A missense variant in *SH2B3* was observed in 4 subjects. This gene is involved in a range of signaling activities by growth factor and cytokine receptors as part of the SH2B adaptor family of proteins. Mutations in this gene have been associated with susceptibility to celiac disease type 13 and susceptibility to insulin-dependent diabetes mellitus. It has low expression in the brain however as evident in the Genotype-Tissue Expression (GTEx) database. Missense mutations in *STIL* were observed in 3 subjects. STIL is a cytoplasmic protein which plays a role in the regulation of the mitotic checkpoint machinery. It too has low expression levels in all GTEx brain tissues.

There are a number of prioritized signals observed in this Saudi epilepsy study that may be novel or have very limited reports of association. Deficiency of *WARS2* was observed in a patient with severe infantile-onset leukoencephalopathy, profound intellectual disability, spastic quadriplegia, epilepsy and microcephaly [39]. Rare mutations in *DPYD* have been implicated in children with unspecific neurological symptoms [40]. Epileptic encephalopathy caused by recessive loss-of-function (LoF) mutations have been reported in *DENND5A* [41]. A previous report of infantile cerebral and cerebellar atrophy showed association with a mutation in *MED17* [42]. A LoF mutation in *HCN4* has been reported to be associated with Familial benign myoclonic epilepsy in infancy [43]. Mutations in *STRADA, SYNJ1, CACNA1A* and *NPRL3* have also been reported with severe epilepsy-related disease, but the association has not been reported for heterozygous variants in isolated forms of epilepsy [44–51].

A number of prioritized signals of putative pathogenicity were observed that have no reports of association with epilepsy in the literature including *SEC24D, PCCA, MYO5A* which may be strong candidates for further functional studies. *SEC24D* was reported to play a role in in vesicle trafficking and mutations in this gene are associated with Cole-Carpenter syndrome, a disorder affecting bone formation [52]. *PCCA* codes for the alpha subunit of the mitochondrial enzyme Propionyl-CoA carboxylase, and mutations in this gene leads to enzyme deficiency and are associated with propionic acidemia [53]. *MYO5A* encodes myosin 5A, and mutations in this gene are associated with Griscelli syndrome, which is characterized by hypopigmentation and a primary neurological abnormality [54]. These aforementioned genes may be good candidates for further functional studies.

This study is limited in that incomplete pedigrees and depth of sub-phenotyping are available, although putative pathogenic variants many known epilepsy-related loci are evident, and a number of potential new loci may be prioritized for further investigation. The Saudi population offers a lot of promise for elucidation of the genetic etiology of common diseases such as epilepsy due to consanguinity, with extended homozygosity stretches often observed over several megabases, affording the opportunity to enrich for recessive forms of epilepsy. While this study looked at 144 subjects, we are aiming to expand the cohort to encompass genetic analyses of extended pedigrees with additional phenotyping.

## Conclusions

We identified for the first time 32 potentially causative pathogenic variants in Saudi individuals with epilepsy. In addition, several potential new loci have been identified that have no reports of association with epilepsy in other populations. These potentially causative pathogenic variants in these new loci may be prioritized for further functional studies.

## Supplementary Information

**Additional file 1.** **Supplementary Table S1:** outlines additional minor allele frequencies in additional databases and further annotation of these putative pathogenic variants.

**Additional file 2.** **Supplementary Table S2:** identified 232 variants of unknown significance across 101 different genes in 133/144 (92%) subjects.

## Data Availability

The datasets generated during the current study are available in the European Nucleotide Archive (ENA)repository, https://eur02.safelinks.protection.outlook.com/?url=https%3A%2F%2Fwww.ebi.ac.uk%2Fena%2Fbrowser%2Fview%2FPRJEB57370&data=05%7C01%7Caalali%40iau.edu.sa%7Cf185a40ee56a4cfbd98808dacb2affab%7C2c86bbfc8d0441ffa83a942f075e0f60%7C0%7C0%7C638045682787922255%7CUnknown%7CTWFpbGZsb3d8eyJWIjoiMC4wLjAwMDAiLCJQIjoiV2luMzIiLCJBTiI6Ik1haWwiLCJXVCI6Mn0%3D%7C2000%7C%7C%7C&sdata=49Th7XeOmQ6HTM69EHDOdReNEmdK7NEb2QrS%2FXWE%2B4w%3D&reserved=0,, under the title" Whole‐Exome Sequencing of a Saudi Epilepsy Cohort Reveals Association Signals in Known and Potentially Novel Loci" with accession number PRJEB57558. All requests for data can be sent to the corresponding author (AKA), and verified academic investigators will be granted full access.
